# Mega Hpv laboratories for cervical cancer control: Challenges and recommendations from a case study of Turkey

**DOI:** 10.1016/j.pvr.2019.03.002

**Published:** 2019-03-13

**Authors:** Murat Gultekin, Mujdegul Zayifoglu Karaca, Irem Kucukyildiz, Selin Dundar, Bekir Keskinkilic, Murat Turkyilmaz

**Affiliations:** a Hacettepe University Faculty of Medicine, Department of Obstetrics and Gynecology, Division of Gynecologic Oncology, Ankara, Turkey; b Dr Zekai Tahir Burak Research and Training Hospital, Ankara, Turkey; c Tokat Research and Training Hospital, Tokat, Turkey; d Turkish Ministry of Health, Public Health Institute, Department of Cancer Control, Turkey

**Keywords:** Turkey, Cervical cancer, HPV DNA, Screening, Mega HPV lab, WHO

## Abstract

Cervical cancer is the fourth most common cancer among women in the world. It is estimated that one woman dies every 2 min from cervical cancer. Nearly all cervical cancers are preventable by early detection and treatment through screening or HPV vaccination. In 2018, World Health Organization (WHO) made a global call for action toward the elimination of cervical cancer. Cervical cancer screening involves a complex organized program, which begins with a call/recall system based on personal invitation of eligible women, followed by participation in screening, and leading to diagnosis, treatment, and management as appropriate. An effective cervical screening program with high coverage is dependent on each country's infrastructure and human resource capacity. Efforts to develop an effective program is particularly challenging in low and middle income countries (LMIC) where resources are limited. For an effective strategy, Turkey redesigned the country's cervical screening program. The local call/recall system and centralized monitoring system of individual women were re-vamped with an automated evaluation system. The revised screening program includes the use of primary HPV testing with a well-defined protocol outlining the algorithms of management (i.e., screening intervals and referral), a single nationwide centralized diagnostic laboratory, and a sustainable agreement with the HPV diagnostics industry. This system allows for traceable, real-time monitoring of screening visits and specimens. Turkey reports on the first four years of this re-vamped organized program and shares lessons learnt from the implementation of this new program.

## 1 Introduction

Cervical cancer is an important public health problem. Globally, cervical cancer ranks as the fourth most common cancer among women with approximately 500,000 new cases and 250,0000 deaths occurring each year; and the burden is disproportionate in less developed countries [1]. The majority of cervical cancers are preventable by early detection and treatment of pre-cancers before they become invasive cancer or by human papillomavirus (HPV) vaccination. In 2018, World Health Organization (WHO) made a global call for action toward the elimination of cervical cancer [2]. Because high-risk oncogenic HPV is the causative agent of nearly all cervical cancer cases (∼99%) [3], prevention strategies that include HPV vaccination and primary HPV testing strategies are essential [4].

Cervical screening involves a complex organized program that begins with a call/recall system based on personal invitation of eligible women, followed by participation of women in screening, and finally leading to diagnosis, treatment, and management as appropriate. An effective cervical screening program with a high coverage is dependent on each country's infrastructure and human resource capacity. Efforts to develop an effective program is particularly challenging in low and middle income countries (LMIC) such as Turkey, where resources are limited and it is difficult for health policy makers to implement effective cervical cancer control strategies.

Conventional screening methods include cytology-based screening with Pap smear, visual inspection with acetic acid (VIA), visual inspection with Lugol's iodine (VILI), and more recently primary HPV testing [5]. Globally, in countries where an organized screening program has been possible, Pap smear is the most commonly used approach and its implementation and clinical experience is well documented [6]. Although centrally organized programs have existed since the 1960s and 1970s, and there is evidence which shows that early detection and treatment of preinvasive lesions can prevent a large proportion of cervical cancers, high coverage has been lower than expected. In the European Union (EU), only 12 countries have successfully implemented a screening program with >70% coverage of the eligible target screening population [7].

Although cytology screening has been available in Turkey for nearly four decades, an organized population-based program was introduced only in 2004 [8]. The organized program has largely been ineffective with coverage rates of 1–2% of the target population, with 40% of screening tests accounted from opportunistic screening between 2004 and 2012 [[9], [10], [11], [12], [13], [14]]. The main reasons for this low population coverage rate is largely explained by the large target population with insufficient human resources (e.g., cytopathology experts) and the absence of quality assurance to ensure that the screening pathway from the call/recall system, cytopathology, colposcopy and histopathology adhered to a rigorous standard [8,[15], [16], [17], [18]]. A large portion of the smears (range between 24% and 75% in different provinces) performed were reported as “insufficient sampling,” or as “infection-inflammation.” In addition, the majority of evaluated Pap smears were reported as normal, and the accuracy of these results were unknown [[9], [10], [11], [12], [13], [14]]. Similar to Turkey, many LMIC countries face similar challenges to develop and sustain an effective cervical screening program. To overcome these challenges, new technologies and investments are needed, especially in LMIC countries, where the burden of cervical cancer is the highest.

Since the causal relationship between HPV infection and cervical cancer was established, HPV tests have been developed and its utility as an alternative modality for cervical cancer screening has been extensively investigated [19]. The accumulated body of evidence shows that HPV testing has a higher sensitivity and higher negative predictive value than pap-smear screening and eliminates the absence of inter- and intra-observer variations that exists in cytology testing, thereby providing more objective results [[19], [20], [21], [22], [23], [24], [25], [26]].

## 2 The new Turkish program for cervical cancer screening

In 2014, Turkey redesigned the cervical screening program including the call/recall system that invites women for screening. The program has a centralized and fully automated monitoring system of individual screening status. The revised screening program includes the use of primary HPV testing with a well-defined protocol outlining the algorithms of management (i.e., screening intervals and referral), a single nationwide centralized diagnostic laboratory, and a sustainable agreement with the HPV diagnostics industry (Mega HPV Laboratory). This system allows for traceable, real-time monitoring of screening visits and specimens. To date, 4 million eligible women have been screened, and the results of the first million women were recently published [27]. However, the details of the laboratory processing and work-flows have not been adequately reported. We further summarize the experiences gathered within the first four years of the program, including the advantages, challenges, and solutions that were identified.

### 2.1 National screening guidelines

In 2012, Turkey launched a new cervical cancer screening guideline [18]. Women aged 30–60 years are eligible for screening. Primary screening with HPV testing every five years is performed by primary-level healthcare staff (i.e., family physicians at population-based screening centers such as KETEM (**K**anser **E**rken Teshis, **T**arama ve **E**gitim Merkezi; Cancer Early Diagnosis, Screening and Educational Centers) staff. Target population between this age interval was almost 16 million for five years and approximately 3 million (16/5) per each year [28].

### 2.2 Screening work flow

In Turkey, there are approximately 24,000 family physicians and nurses in the entire country who can perform screening. Each family physician and their nurses care for approximately 3,500 women in the target population, which equates to 800–1000 women being screened in a five-year period (ie.,150–200 women per year). They are responsible for keeping the patients’ records up-to-date by using a specific National Screening Software system, called RUNLEK, for call and recall. Eligible women can be invited via e-mail, telephone, face-to-face interviews, or through letter invitations. If there is no response, a new invitation is resent annually, and if there is non-attendance after five consecutive years, a “rejected screening” response is recorded [29].

**Fig. 1:**
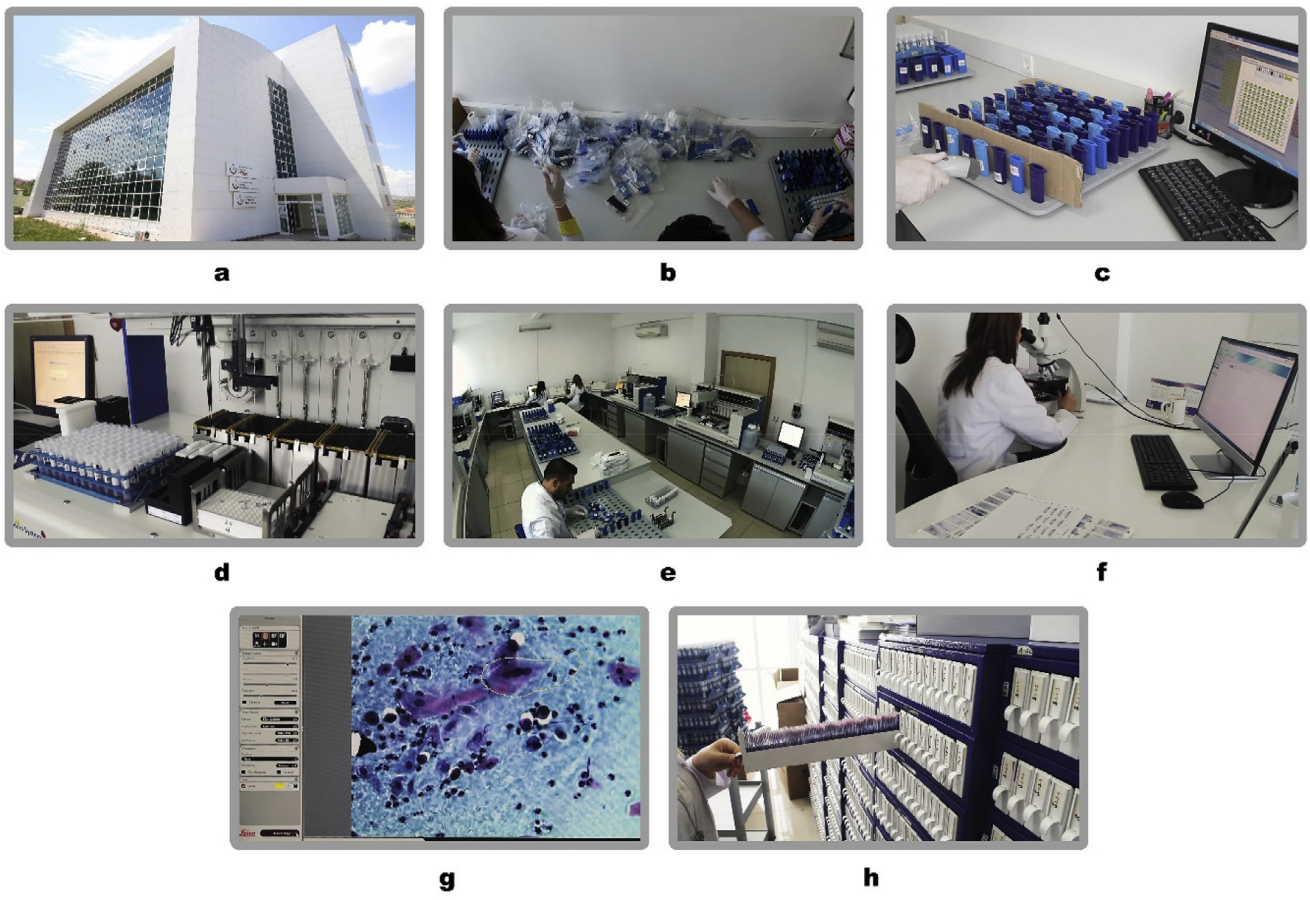
Flowchart of the National HPV Laboratory. 1a National HPV Lab is on Floor 2 with 7 Rooms, 1b Two barcoded sample from the same patient (conventional cytology and HPV DNA) arrives to the laboratory by Cargo, 1c Barcoding system and software does not allow any mismatch among the millions of samples received 1d Rapid Capture System (RCS) by Qiagen ^®^ 1e 10 RCS System works simultaneously to detect HPV DNA 1f -1g Cyto-pathologists evaluating the pap Smears in a double blind manner and saving the digital pictures (cyto-gallery) 1h Storage of the positive (5 Years) and negative samples (3 months).

At screening, two samples are taken from each woman. This enables cytology testing for those who are found to be HPV-positive without the need for the woman to return for a separate clinical visit to provide an additional sample for cytology testing. The first sample is collected with a brush and transferred to a glass slide for conventional cytology. The second sample is taken with a different brush and placed in 5 ml of Standard Transport Medium (STM) for HPV DNA analysis. For women who are HPV positive by Hybrid Capture2 (Qiagen), genotyping is performed with the CLART kit (Genomica) (Fig. 1).

The HPV and cytology sampling kits are prepared by the national central laboratories, where kits are barcoded and distributed to primary health care centers which allow tracking and monitoring of kits from RUNLEK, which is an online web-based platform. Following the collection of samples, samples are stored at each local center and sent weekly to the central HPV laboratory. By taking two samples, patients with an HPV-positive test will have their cytology sample automatically tested without recall. This allows for a more cost-effective approach in which resources are focused on increasing cervical cancer attendance and follow-up of women who require treatment and management.

### 2.3 National HPV and cytology laboratory

Two national laboratories were established for the primary HPV screening program in Ankara and Istanbul (Fig. 1). A total number of 32 staff members work in these two laboratories which include 4 pathologists and 2 microbiologists, who are responsible for managing the laboratories. Each week 65 provinces send their samples to the Ankara laboratory, and 16 provinces send their samples to Istanbul. All specimen testing are performed in the two laboratories by using fully automated operational procedures that allow tracing of specimens and timely delivery of results to the screened population. Each laboratory has the capacity to manage 30,000 samples and testing per week and has the capacity to be upgraded to handle more samples if needed. Currently, about ten Rapid Capture Systems (RCS-Qiagen ^®^) are sufficient to manage the weekly screening numbers. Each system can carry out two to three runs per a day with a capacity to load 384 samples per run and 9,400 samples daily. All the samples received by the laboratory are processed, tested, and reported within 10 working days. The time needed to review and report a woman's result is estimated to be 6 h for HPV-negatives and 15 h for HPV-positives. These laboratories are uniquely set-up to handle this high volume of molecular diagnostics testing each day. According to the number of women attending each week, the number of runs or the number of RCS devices can be easily increased if required.

For patients who are HPV DNA positive, two pathologists are double-blinded and evaluate the conventional cytology samples. As there are only four pathologists across the two laboratories, Pap-smears of HPV positive cases are evaluated with uniformity and low inter-observer variability. The four pathologists also evaluate each other to ensure quality assurance (Fig. 1). For example, if one pathologist reports 10% of slides are normal (NILM), a second pathologist will re-review the slides to ensure inter-observer consistency and quality control. The National Screening software system enables the pathologists to share digital images of suspicious smears across the two laboratories and enable health directors to also carry out a double-blinded review of the four pathologists.

HPV-negative samples are stored for 3 months, whereas HPV-positive samples are stored for 5 years with the cytology slides and in the patient's digital records (cyto-gallery) (Fig. 1). This National Screening software system allows all patients to have a copy of their smear and HPV testing results at any time they require, and both patients and physicians are able to retrieve the data as needed.

As HPV testing represents a new technology used in the program, quality assurance is necessary to ensure accuracy of results. For internal control, one negative and one positive HPV specimen are used for each 88-sample plate that is run. To ensure external quality control, a number of samples are distributed to the UK National External Quality Assessment Service (UK NEQAS) three times a year for repeat testing.

This HPV technology provided the feasibility of upscaling a primary screening program and helped inform Turkey's health policy makers and their decision-making process. The flexibility of the screening system that could be upgraded and adapted to the needs of the screening program without the limitation of human resources (cyto-technician or pathologist) supported the implementation of this redesigned program. The infrastructure and workflow of the National HPV Lab can be viewed at www.youtube.com/watch?v=IBmAflRjI10. A full HPV genotyping map of the entire country was also achieved at the end of the four-year implementation period, which will help to inform the future direction of cervical cancer control including HPV vaccination.

### 2.4 National Screening Software

RUNLEK (www.runlek.com), a specific National Screening Software, was developed to support the redesigned population-based primary HPV screening program. The system allows for monitoring of each step of the screening process. The software can track starting from the HPV kit distribution process to the centers, to sample collection and logging of samples at the center, transport of the collected samples, and finally, testing, review, and reporting of results. If there is a problem/error at any stage of the process, a warning message is sent to the laboratory directors; thus, it is possible to prevent, detect, and resolve the problem.

Results are reported online and sent to each physician/nurse once they are available. Medical staff are also able to access the results by logging into the online platform. Results include adequacy of the samples, HPV positivity vs. negativity, HPV Genotypes for positive cases (16, 18, 31, 33, 35, 39, 45, 51, 52, 56, 58, 59, 68, and other) and cytological abnormalities for those HPV-positive. In addition, patients also have the facility to access their results thorough a web-based platform by using the barcode numbers and personnel citizen numbers (https://hpvtarama.saglik.gov.tr/duyurular/sonucsorgula).

RUNLEK is also able to generate reports for health authorities about HPV positivity, screening rates, HPV genotypes and cytological abnormalities, and overall scenario, according to health center.

### 2.5 Post-screening centers

The Ministry of Health implemented at least one post-screening diagnostic center for each of the 81 provinces in Turkey. If required, patients are treated and managed within two months following HPV-positive and cytology positive results. Specifically, the ministerial guidelines refer women to colposcopy if she is HPV-16/18 positive with abnormal cytology. Each center has at least two gynecologists. All services from screening to diagnosis and treatment is provided free of charge. When the laboratory results are sent back to the physicians, a secretarial telephone number is also provided for positive cases so that a colposcopy appointment can be scheduled immediately. All diagnostic centers are reviewed twice a year to evaluate the number of patient referrals, number of patients who obtained an appointment, number of colposcopies performed, number of punch biopsies or LEEP procedures and the results of final pathologies. Although RUNLEK integrates the data from primary screening to the HPV and cytology results, data from the diagnostic centers are not integrated into this central screening software system but will be collected by the cancer registry units in each of the 81 provinces.

## 3 Advantages of the new screening program

### 3.1 General screening rates

Since introduction of the new primary HPV screening program, screening coverage and uptake on population-based program has increased up to ten-fold from 3% in 2012 to 35% in 2017 (18, 27). These data show that high screening coverage can be achieved in a predominantly Muslim population. Good coverage was also achieved in rural vs. urban areas, homogeneously across the entire country. Based on surveys of General Practitioners, there was ∼36.5% acceptance rate for HPV-based cervical cancer screening after first invitations. This rate was 63.5% for ages 30–45 years, 32.7% for ages 45–60 years, and 13.5% for ages 60 years and older. The attendance rate among those who accepted the first invitation was 82.8%. The most common invitation method was by telephone including SMS (60% of the invitations), followed by face‐to‐face invitations (30% of invitations). These had a higher acceptance rate (approximately 80–90% for telephone). For the remaining 10% of women, especially in highly populated provinces which has a higher incidence of young and working population, letters, leaflets, brochures, or social media were used for invitations, but the acceptance rates were lowest for these methods, being approximately 30–40% (27). Although there was limited success with an effective cytology-based screening program, the change in method of screening and the re-designed program has motivated women to attend screening without any significant effort in mass media campaign or cancer educations compared to previous years.

### 3.2 Human resources (cyto-pathology and colposcopy experts)

Of the total number of 3.8 million women screened in years 2014–2018, HPV positivity rate was around 4.29%. Among them, 30.9% were found to have abnormal cytology (1.32% of HPV positives). With HPV DNA screening, only 4% positivity has decreased the number of re-visits for re-samplings. The low positivity rate of HPV DNA in Turkey indicated that HPV DNA screening program could be a choice while implementing population-based cervical cancer screening system, especially in countries with similar low HPV prevalence rates.

The new screening system provided us a big opportunity and advantage in colposcopy referral. Given the low rate of HPV positivity (4%) plus the triage by reflex cytology and full genotyping resulted in a 1.3% colposcopy referral rate [27]. Such a low rate did not bring additional colposcopy device burden for the Ministry of Health. Additionally, continuous medical colposcopy practice trainings were organized centrally by the Ministry of Health, 2–3 times a year; this helped in networking, communication, and improving the skills of the gynecologists who are working in the referral colposcopy centers.

### 3.3 Future HPV policies

Another advantage of the new screening program was to enable to undertake genotyping of the entire country, even by street by street. Even if the HPV DNA is low in prevalence, genotype distributions showed characteristics of a bridge between Asia, Africa and Europe having number one HPV type of each continent in top five HPV genotypes of Turkey [27,30]. This would definitely bring valuable information for the future vaccine policies of the countries.

## 4 Challenges and solutions with the new screening program

### 4.1 Cyto-pathology advocates

There were different kinds of challenges faced during realization period of the new screening project, which started with the big debates on screening via pap smear vs HPV DNA tests within the Turkish cyto-pathology groups. The national screening data were evaluated in ministerial workshops by Turkish Societies of Gynaecological Oncology, Colposcopy and Cervical Pathology, Clinical Microbiology, and Turkish Society of Cytopathology in order to conclude all the debates. Some international opinions were taken from IARC, ESGO, and key opinion leaders of the literature. Official responses of all stakeholders were collected, and accordingly, new cancer screening guidelines were published by the Ministry of Health that is publicly available at the web page of cancer control department.

### 4.2 Primary health care staff

After the new screening system started, another problem encountered was the resistance of the primary level health staff, family physicians and their nurses, which was regarding the excess work burden on them. Screening interval of 5 years provided by HPV DNA made it easy for many family physicians and their nurses to achieve a 100% coverage and planned invitations, compensating the extra work burden of cancer screening compared to pap smear with more frequent annual intervals. The RUNLEK program enabled all screenings performed anywhere in the country to be accessible to the family physician. Therefore, even if the patients were screened in KETEMs or different primary level health structure, her name was dropped off from the target list for screening in her family physician's computer record.

Another problem was many of the family physicians were unaware and untrained about HPV, sample collection, and informing HPV-positive patients. To meet this need, the Ministry of Health organized several workshops with the Federation of Family Physicians (AHEF) in different regions of the country. Videos prepared for sampling were sent digitally to all primary health stuff. All screening staff were also specially trained for communication and HPV screening through video training modules prepared by the Ministry of Health. These trainings are being continued as a refresher education in a standardized way, 3–4 times a year.

There were also some challenges met during the operational process. First; there was no national HPV vaccination program, but raising HPV awareness consequently brought increased interest in HPV vaccines. Particular concerns and questions were about adult vaccination, vaccination of the householders, and effect of vaccines on already HPV positivity.

### 4.3 Gynecology and colposcopy experts

Another problem faced was the inadequate training of the gynecologists for colposcopy and lack of a quality assurance system and data retrieval system within the colposcopy units. Even if the total number of colposcopies around the entire country was sufficient and there was no queue for colposcopy, the gynecologists had unwillingness for performing the procedure due to malpractice fears. The Ministry of Health has organized several colposcopy workshops (3–4 per year) throughout the country but still data retrieval and quality are distant from the universal targets. Further, some of the gynecologists did not follow ministerial guidelines for primary HPV screening and algorithms for diagnosis, treatment, and management. Instead of a direct colposcopy for patients referred to them (HPV positive cases with abnormal cytology/HPV 16 or 18 Positive cases), 40.9% of the patients were evaluated by a repeat smear or a repeat HPV test. (27) In some of the patients, the repeat HPV test was discordant with the first result because of technical differences between the methods. Such discordant results also caused a confusion among the attending people.

## 5 Conclusions

Primary HPV DNA screening is feasible in many countries with a single Mega HPV laboratory. It is more objective, automatized, and has no inter- or intra-observer variability. With a crowded population targeting screening, it is even cheaper than cytology, with additional advantages of extended screening intervals and higher sensitivity and negative predictive value. The results have shown that the attention and participation of females are high even in developing and conservative countries such as Turkey. Low HPV prevalence is also an advantage for similar profile countries giving a 4% need for reflex cytology evaluations. Our country experience shows that besides these advantages; if the countries that plan to implement similar systems become aware of the challenges faced during the initial phases of the screening, HPV DNA tests would be appropriate choice for implementing nationwide population-based cervical cancer screening programs.

## Conflicts of interest

The authors declare that they have no conflict of interest.
